# Intranasal Transplantation of Microbiota Derived from Parkinson’s Disease Mice Induced Astrocyte Activation and Neurodegenerative Pathology from Nose to Brain

**DOI:** 10.3390/brainsci15050433

**Published:** 2025-04-23

**Authors:** Yi-Meng Xia, Mei-Xuan Zhang, Xiao-Yu Ma, Lu-Lu Tan, Ting Li, Jian Wu, Ming-An Li, Wei-Jiang Zhao, Chen-Meng Qiao, Xue-Bing Jia, Yan-Qin Shen, Chun Cui

**Affiliations:** 1 Laboratory of Neurodegenerative Diseases, Wuxi School of Medicine, Jiangnan University, Wuxi 214122, China; 6222803012@stu.jiangnan.edu.cn (Y.-M.X.); 6212803015@stu.jiangnan.edu.cn (M.-X.Z.); 6222809046@stu.jiangnan.edu.cn (X.-Y.M.); 6222803008@stu.jiangnan.edu.cn (L.-L.T.); 7212808006@stu.jiangnan.edu.cn (T.L.); 7202808007@stu.jiangnan.edu.cn (J.W.); 7232808017@stu.jiangnan.edu.cn (M.-A.L.); weijiangzhao@jiangnan.edu.cn (W.-J.Z.); qiaochenmeng@jiangnan.edu.cn (C.-M.Q.); 8201906124@jiangnan.edu.cn (X.-B.J.); shenyanqin@jiangnan.edu.cn (Y.-Q.S.); 2 MOE Medical Basic Research Innovation Center for Gut Microbiota and Chronic Diseases, Wuxi School of Medicine, Jiangnan University, Wuxi 214122, China

**Keywords:** Parkinson’s disease, nasal microbiota, nose-brain axis, olfactory dysfunction, astrocyte activation

## Abstract

Background: Parkinson’s disease (PD) is characterized by early-onset olfactory dysfunction preceding motor symptoms, yet its mechanisms remain elusive. Based on the studies on microbiota-gut-brain axis, the microbiota–nose–brain axis might be involved in the pathogenesis of PD. However relative studies are rare. Methods: By consecutive 14-days intranasally transplanting bacteria, we established mice models exhibiting nasal microbiota dysbiosis (NMD), including animal group received intranasal drops of fecal bacterial suspension from normal mice (NB group) and animal group received intranasal drops of fecal bacterial suspension from PD mice (PB group), with animals that only received anesthesia used as the control group. Then we analyzed the nasal microbiota composition via 16S rRNA sequencing, evaluated the olfactory and motor functions through behavioral experiments, including buried food test, open field test, pole descent test, and traction test. The neuropathology in olfactory-related and PD-related brain regions, including olfactory bulb, pyriform cortex, hippocampus, substantia nigra and striatum, was also detected by western blotting, immunofluorescence and immunohistochemical experiments using the antibodies of NeuN, TH and GFAP. Results: 16S rRNA sequencing revealed that PB mice were primarily characterized by an increase in bacteria associated with inflammation and PD. Behavioral assessments revealed that mice with NMD demonstrated impairments in the buried food test and pole descent test, indicative of olfactory and motor dysfunction. By detecting NeuN and GFAP expression, we identified neuronal loss and astrocytes activation in olfactory-related brain regions and adjacent structures, including the olfactory bulb, pyriform cortex, hippocampus, substantia nigra and striatum of both NMD groups, which may contribute to the observed functional disorders. Notably, animals exposed to PD-derived bacteria exhibited more pronounced changes in nasal bacteria, with more severe neuropathology. Conclusions: We present evidence supporting the microbiota–nose–brain axis, and the NMD-induced astrocyte activation and neurodegenerative pathology along the olfactory pathway may serve as a link between nose and brain.

## 1. Introduction

Parkinson’s disease (PD) is the second-most prevalent neurodegenerative disorder globally, with the global number of PD patients projected to reach 25.2 million by 2050, representing a 112% surge in prevalence since 2021 driven, primarily by population aging [1]. Therefore, new investigations into the pathophysiology of PD are critical, facing the increase in life expectancy and the lack of effective pharmacological treatments for PD progression. PD patients manifest typical motor symptoms, such as resting tremor, bradykinesia, myotonia, postural and gait abnormalities, which are mainly due to the massive death of dopaminergic neurons in the substantia nigra (SN) leading to a decrease in dopamine projected to the striatum [2]. The pathogenesis of PD is related to age, genetic or environmental factors [2] and involves multiple pathological mechanisms, including abnormal aggregation of α-Syn, oxidative stress injury, apoptosis, neuroinflammation and so on, among which, neuroinflammation mainly regulated by glial cells is considered to be one of the main causes [3].

Interestingly, PD patients usually show non-motor symptoms before the onset of motor symptoms, such as constipation, sleep disorders, depression, autonomic dysfunction [4], olfactory dysfunction [5], subjective cognitive decline and mild cognitive impairment [6]; among them, olfactory and gastrointestinal dysfunction is particularly common. About 90% of PD patients have experienced olfactory dysfunction for several years preceding the onset of motor symptoms [7]. Therefore, olfactory dysfunction is regarded as an important early sign of PD. Actually, in several types of neurodegenerative diseases, such as Alzheimer’s disease, Huntington’s disease, multiple sclerosis, etc., olfactory dysfunction is also considered as a clinical early-stage marker [5]. However, the relation of olfactory dysfunction with the brain disorder remains unclear even now.

Olfactory dysfunction is usually associated with nasal infection or nasal inflammation [8,9]. Several studies also confirmed that nose-originated inflammation may “transfer” to the brain and eventually produce neurological damage or brain-related malfunction; a mouse model of chronic rhinitis by localized overexpressing TNF-α in the olfactory epithelium exhibited olfactory dysfunction and brain-controlled behavioral abnormalities of social behaviors and gustatory impairments [10]. In mice with allergic rhinitis induced by ovalbumin, the inflammatory factor levels were elevated in the hippocampus, with disturbed spatial learning memory [11]. Thus, it can be seen that systemic or CNS-specific inflammation might be a bond between the nose and brain.

Nasal bacteria can mediate nasal lesions and olfactory function through inflammation caused by the direct translocation of bacteria or their metabolites [8,12,13,14,15]; therefore, inspired by the studies on the microbiota–gut–brain axis [16], the microbiota–nose–brain axis is supposed to play an important role in neurodegenerative disease [17], in which nasal microbiota is supposed to affect the CNS through the olfactory pathway (including olfactory epithelium, olfactory bulb (OB), pyriform cortex (PC), amygdala, the hippocampus, etc.) or the trigeminal nerve pathway [18]. However, there are no studies to confirm this hypothesis.

The microbiota–nose–brain axis might also function in PD, despite several studies finding no significant difference in the nasal bacterial composition of PD patients from the normal population [19,20], which might be due to the variable depth of nasal microbiota sampling and the high environmental vulnerability of the open nasal cavity. While collecting nasal microbiota with deep nasal swabbing under nasal anterior endoscopy, Pal et al. reported deep nasal microbiota dysbiosis in PD patients, characterized by an increased abundance of phylum *Proteobacteria*, especially *M. catarrhalis* [21], which is commonly accepted as bacterium with inflammatory properties [22,23]. As mentioned before, most PD patients have experienced olfactory dysfunction, and the volume of OB is significantly reduced in PD patients [24]. According to the Braak’s PD staging hypothesis, the OB and the gut may be the initial pathogenic sites of PD [25]. In the case of OB origin, α-Syn aggregation begins from the OB and progressively travels up to the anterior olfactory nucleus, the PC, the amygdala, the hippocampus and the SN to the other brain regions of PD patients [25]. MPTP-induced PD mice also exhibited olfactory deficits [26] and inflammation along the olfactory pathway [27]. Based on these findings, the microbiota–nose–brain axis might be a critical mechanism in PD, but further study is needed to prove it.

To validate the microbiota–nose–brain axis hypothesis in PD, intranasal transplantation of deep nasal microbiota derived from PD patients or animal models represents the optimal approach. However, this methodology faces dual feasibility challenges: not only is deep nasal microbiota collection from human PD patients clinically problematic, but the procedure also necessitates sacrificing substantial numbers of experimental animals due to the limited microbial biomass obtainable from individual specimens through nasal turbinate tissue harvesting. Then, PD-derived fecal microbiota was used as a practical alternative given the current sampling limitations. Because, on the one hand, the pathogenesis of PD begins from both the nose and gut, there is the possibility of nasal contamination by intestinal microbiota; on the other hand, the fecal bacteria of PD mice showed definite PD pathogenicity, we supposed PD-derived fecal bacteria treatment induced more severe pathological or behavioral changes compared to normal fecal bacteria treatment [28]. Therefore, we generated mice with PD-specific nasal microbiota dysbiosis by intranasal transplantation of PD fecal bacteria. We hypothesize that the intranasal administration of pathogenic bacteria will induce nasal microbiota dysbiosis, which might further induce neurodegeneration and glial activation along the olfactory pathway and adjacent brain regions, like the SN and striatum, that will lead to olfactory and motor dysfunction in mice. Based on these hypotheses, we employed 16S rRNA sequencing to analyze the diversity and composition of nasal microbiota in mice and assessed neuronal loss and glial activation in the brain structures along the olfactory pathway (including OB, PC and hippocampus) and SN and striatum. Thus, it might provide evidence for the hypothesis of the microbiota–nose–brain axis; nasal microbiota dysbiosis can induce both olfactory and cerebral dysfunction, and the migration of inflammation along olfactory pathway trigged by the nasal microbiota dysbiosis might play important role in this crosstalk.

## 2. Materials and Methods

### 2.1. Animals

All 6-week-old male mice of C57BL/6J used in this study were purchased from the Vital River Laboratory Animal Technology Co., Ltd., Beijing, China. These mice were transported to the Medical Laboratory Animal Center of Jiangnan University, where they first completed a 7-day environmental acclimatization period in the animal quarantine isolation area. Subsequently, they were transferred to a specific pathogen-free (SPF) compliant facility for subsequent experiments. During the rearing period, the ambient temperature was maintained at 24 ± 2 °C, humidity was controlled at 55 ± 10% and a standard daylight cycle was followed. The experimental animals were entitled to clean drinking water and standard formulated feed at all times. All experimental procedures involving animals firmly followed the guidelines and codes of practice set by the Animal Welfare and Ethics Committee of Jiangnan University (Approval number: JN.No20220530c0601231(162)).

### 2.2. Preparation of PD-Derived Fecal Bacteria

First, 20–30 pellets of fresh feces from either normal mice (intraperitoneally injected with saline) or subacute PD mice (intraperitoneally injected with 30 mg/kg MPTP once daily for 5 consecutive days) on the 7th day after modeling [29] were collected, respectively, dissolved in sterile PBS (1 pellet of feces/mL PBS) and shaken for mixing, then centrifuged at 2000 rpm for 10 min. The supernatant was collected and centrifuged at 8000 rpm for 5 min, and then, the precipitate was remixed in an appropriate amount of sterile PBS (1 pellet of feces/mL PBS) and added with an appropriate amount of sterile glycerol to the final concentration to 20% and stored in a refrigerator at −80 °C. Before use, diluted smear plates (serial dilution factor: 10–10^9^) were counted using the CDC anaerobic blood agar medium, and the final concentration of the bacterial solution was diluted to 10^8^ CFU/mL with PBS, according to the counting results [30].

### 2.3. Microbiota Intranasal Transplantation and Animal Groups Design

Animal groups design: 7-week-old male C57BL/6J mice were randomly divided into three groups. All these three groups of mice were anaesthetized by inhaling 4% isoflurane via the rodent anesthetic device (SOARMED, MSS-3). Then, they were subjected to different intranasal transplantation of microbiota as follows: (1) Con group: anesthesia only (n = 11); (2) NB group: receiving intranasal drops of fecal bacterial suspension from normal mice (n = 11); (3) PB group: receiving intranasal drops of fecal bacterial suspension from PD mice (n = 11).

The intranasal bacterial drops treatment [31]: Bacterial suspension was sucked up with a pipette gun and carefully dripped into the nostrils of the anesthetized mice drop by drop, which meant that, when one drop was completely inhaled by the mice, the next drop was dripped, with a total volume of 10 μL. Intranasal microbiota transplantation was performed once daily for 14 days.

### 2.4. Behavioral Experiments

Buried food test: We used the buried food test to test the olfactory function [32]. Before the test, mice were fasted for 12 h. A clean standard cage with 3 cm of fresh bedding was used as the test box. Mice were acclimated to the test box for 5 min, then moved to an empty box. Standard feed was then buried in a random corner of the test box, and the mouse was placed in the center to start the timer. Timing stopped when the mouse found and dug out the feed (300 s max). Each mouse was tested five times with 5-min intervals, and the bedding was replaced to avoid odor interference. The average time of the five tests, excluding the highest and lowest values, was used as the score. The whole process was randomized using a double-blind design.

Open field test: This test is commonly used to evaluate the motor behavior, exploratory behavior and anxiety behavior of experimental animals in an open environment [33]. Specifically, mice were placed in an open field (50 cm × 50 cm) with their heads facing the center of the open field, and then, their movement in this open field within 2 min was recorded using EthoVision XT software (version 10.0), including the total distance, average speed and number of squares crossed.

Pole descent test: This test is used to assess the degree of motor retardation in mice [34]. The pole used was 55 cm long and 1 cm in diameter, wrapped with non-adhesive gauze for a better grip. The bottom end was anchored in a cage with bedding. During testing, mice were placed with forelimbs at the pole top, and the time was recorded from placement until their hind limbs left the pole. Each test was repeated three times with a 15-min interval, and the average time was used for analysis.

Traction test: The traction test was performed to assess balance and muscle strength in mice [35]. The rope had a diameter of five millimeters. The mice were scored in the following ways: 4 points were awarded when they used all of their limbs to grasp the rope, 3 points for using two forelimbs and one hindlimb, 2 points for utilizing both forelimbs and 1 point for using just one forelimb. The test was administered three times, with a 15-min break in between. The three mean scores were calculated, and statistical analysis was performed.

The buried food test and open field test were conducted on the day before sample collection; the pole descent test and traction test were conducted on the sample collection day.

All animals in the pole descent test and traction test conducted behavioral pretraining once daily from the 12th day of intranasal dripping for 3 days before the formal test. We conducted behavioral tests with at least 8-h intervals between different tests to minimize the stress effects. The whole process was randomized using a double-blind design.

### 2.5. Sample Collection and Tissue Preparation

After 14 days of intranasal microbiota dropping, on the 15th day, the mice were deeply anesthetized with 4% isoflurane to reduce their pain, then were sacrificed for different uses.

Collection of fresh brain tissues: The brain tissues were rapidly removed, and the OB was rapidly separated and loaded into the corresponding EP tubes, which were immediately put into the ultra-low temperature refrigerator at −80 °C for storage.

Collection of fresh nasal turbinate tissues: For 16S rRNA gene sequencing of deep nasal microbiota, fresh nasal turbinate tissues should be collected. According to Zeppa’s method [31], after the collection of brain tissues, the heads of the mice was further removed from the mandible, tongue and other tissues, and then, the palate was torn off to expose the molars, then the molars were gently broken with curved scissors to the side, exposing the turbinate tissues at the back. Then, the turbinate tissues were carefully collected into the cell cryotube with sterile tweezers and then put into liquid nitrogen to be frozen and stored in the refrigerator at −80 °C.

Tissue preparation for immunofluorescence (IF) or immunohistochemistry (IHC) analysis (n = 3/group): The mice were perfused with phosphate-buffered saline (PBS) transcardially, succeeded by 4% paraformaldehyde (PFA). Subsequently, the whole brain was extracted and fixed in 4% PFA at 4 °C overnight, then incubated in 20% sucrose for 24 h, followed by 30% sucrose for another 24 h at 4 °C, and ultimately embedded in the Optimal Cutting Temperature (O.C.T.) compound (Tissue-Tek, Torrance, CA, USA). Coronal sections of 10 μm in thickness were then prepared using a CM1950 cryostat microtome (Leica, Wetzlar, Germany).

### 2.6. 16S rRNA Gene Sequencing for Nasal Microbiota

The frozen mice nasal turbinate tissues were sent to Novogene (Beijing, China) for DNA extraction and 16S rRNA gene sequencing. The microbial genomic DNA was extracted from the nasal turbinate tissues using the CTAB (hexadecyl trimethyl ammonium bromide) method, and the 16S V4 region was amplified using specific primers with Barcode. The primer sequences were GTGCCAGCMGCCGCGGTAA and GGACTACHVGGGTWTCTAAT. The PCR amplification of the V4 region of the bacterial 16S rRNA gene was carried out under the following conditions: The first denaturation was carried out at 98 °C for 1 min, followed by 30 cycles at 98 °C (10 s), 50 °C (30 s) and 72 °C (30 s) and then a final cycle at 72 °C for 5 min. The PCR products were purified by the Universal DNA Purification and Recovery Kit (Tian Gen, Beijing, China), and the library was constructed using the NEB Next^®^ Ultra™ II FS DNA PCR-free Library Prep Kit (New England Biolabs, Ipswich, MA, USA), and the constructed library was quantified by Qubit and q-PCR. The constructed library was quantified by Qubit and q-PCR, and after the library was qualified, NovaSeq 6000 was used for PE 250 online sequencing. The data obtained from the assay were clustered and analyzed using Uparse software (version 7), and the tagged sequences with the highest abundance in each cluster were selected as representative sequences. Qiime software (version 2) was applied to calculate the α-diversity-related metrics (Chao1, Shannon and Observed-otus) and β-diversity-related metrics (PCoA).

### 2.7. Immunohistochemical and Immunofluorescent Staining

For IHC staining, coronal sections of the OB and midbrain were immersed in 0.01 M sodium citrate buffer (pH 6.0) for antigen retrieval at 95 °C and washed thrice by PBS. Implement the Universal Two-Step Detection Kit (Mouse/Rabbit Enhanced Polymer System) (PV-9000, ZSGB-Bio, Beijing, China) alongside the AEC Enzyme Substrate Kit (ZLI-9036, ZSGB-Bio, China), adhering to the detailed protocol outlined in the reagent manuals. Incubate with the primary antibody of mouse anti-GFAP (1:1000, GB12096-100, Servicebio, Wuhan, China). Conclude with counterstaining using hematoxylin (AR0005, Boster, Wuhan, China) and routinely mounted. Images were captured with a Nikon Eclipse 80i microscope (Kyoto, Japan) and quantified with ImageJ (version 1.8.0) software.

For IF staining, brain coronal sections underwent antigen retrieval by immersion in 0.01 M sodium citrate buffer (pH 6.0) at 95 °C, followed by three successive washes with PBS. Additionally, the sections were incubated in PBS containing 0.3% Triton X-100 and 10% goat serum for 30 min at 37 °C to permeabilize and block non-specific binding sites. Primary antibodies, including rabbit anti-NeuN (1:500, ABN78, Sigma-Aldrich, St. Louis, MO, USA), mouse anti-GFAP (1:1000, GB12096-100, Servicebio, China), mouse anti-Iba-1 (1:200, GB12105-100, Servicebio, China), mouse anti-TH (1:1000, GB12181-100, Servicebio, China) and rabbit anti-TH (1:500, AB152, Millipore, Burlington, MA, USA), were incubated overnight at 4 °C. The following day, secondary antibodies were coupled with Cy3-labeled Goat Anti-Mouse IgG (H+L) (1:1000, Beyotime, Shanghai, China) and FITC-labeled Goat Anti-Rabbit IgG (H+L) (1:1000, Beyotime, China) for 1 h at 37 °C. DAPI was employed to counterstain the nuclei. Fluorescent images were captured using a Zeiss Axio Imager Z2 microscope (Jena, Germany). ImageJ (version 1.8.0) was used to analyze the cell counts in three representative sections (space 100 μm between) of the same region from each mouse, with three mice per group, and the cell number was calculated as the cell density.

### 2.8. Western Blotting

Total protein was isolated from 20 mg of the fresh tissue homogenized in 200 μL RIPA lysis buffer (Beyotime, China) containing 1% phenylmethanesulfonylfluoride (PMSF) (Beyotime, China) and 2% phosphatase inhibitor (Beyotime, China). The homogenate was centrifuged at 13,000 rpm under 4 °C for 5 min. The protein concentration in the collected supernatant was quantified using a BCA Protein Quantification Kit (Vazyme, Nanjing, China). The proteins were then denatured by heating in SDS sample buffer at 100 °C for 15 min, and 30 mg of the total protein were separated on 10% and 12% SDS-PAGE gel, transferred to PVDF membranes (Millipore, USA) and subsequently blocked with 5% skim milk at room temperature for 2 h before incubated with specific antibodies overnight at 4 °C. The following primary antibodies were used to probe the proteins: mouse anti-GFAP (1:1000, GB12096-100, Servicebio, China) and rabbit anti-GAPDH (1:1000, 10494-1-AP, Proteintech, Wuhan, China). Goat anti-mouse IgG (1:8000, BA1051, Boster, China) and goat anti-rabbit IgG (1:8000, BA1054, Boster, China) conjugated with horseradish peroxidase were used as the secondary antibodies. Protein bands were detected following incubation with BeyoECL Plus (P0018, Beyotime, China) for 1 min; after which, they were visualized using a Tanon 5200 Gel Image System (Shanghai, China). Densitometric analysis was conducted using ImageJ software.

### 2.9. Statistical Analysis

Data are expressed as the mean ± standard error of the mean (SEM). Statistical comparisons between the three groups were conducted using one-way ANOVA (one-way analysis of variance) and Tukey’s post hoc test with SPSS 26.0. A *p*-value of less than 0.05 was deemed to denote statistical significance. All statistical evaluations were performed by GraphPad Prism software, version 8.0.

## 3. Results

### 3.1. Intranasal Transplantation of Fecal Microbiota Led to Disorganized Nasal Microbiota in Mice

**Figure 1:**
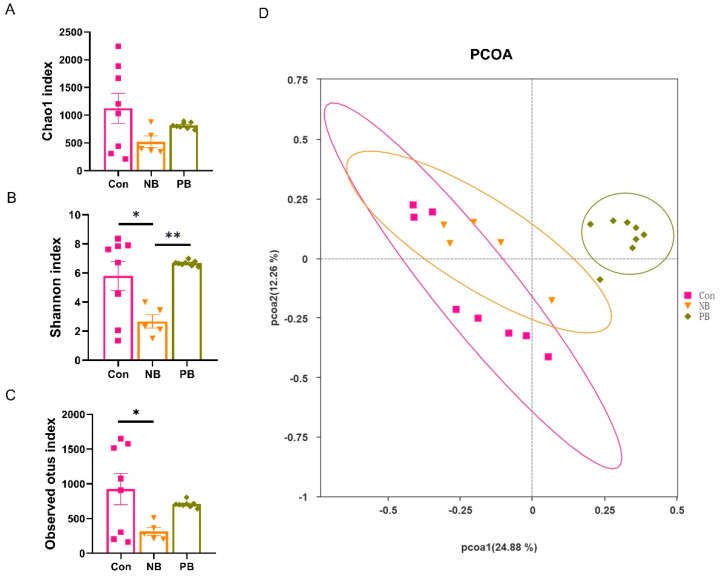
Results of α-diversity and β-diversity analyses of nasal microbiota of mice in each group. (**A**) α-diversity analysis between three groups using the Chao1 index. (**B**) α-diversity analysis between three groups using the Shannon index. (**C**) α-diversity analysis between three groups using the Observed-otus index. (**D**) Principal coordination analysis (PCoA) based on the unweighted unifrac distance algorithm. Data are expressed as the mean ± SEM, and significance was tested using one-way ANOVA with Tukey’s post hoc test. * *p* < 0.05 and ** *p* < 0.01 (n = 5–8/group).

In order to detect whether the 14 days of intranasal transplantation of fecal microbiota altered the composition of the nasal microbiota, we analyzed the microbiota composition of the mice nasal turbinate tissues using 16S rRNA sequencing on the 15th day. The α-diversity analysis reflects the richness and diversity of the nasal microbial community, which can be measured by different indices, including Chao1, Shannon and Observed-otus (Figure 1A–C). Our results showed that there was no significant difference in the Chao1 index among the three groups (Figure 1A). For the Shannon index, the NB group was significantly lower than the other two groups (vs. Con *p* = 0.0188 and vs. PB *p* = 0.0031); there was no difference between the Con group and PB group (Figure 1B). For the Observed-otus index, the NB group was also significantly lower than the Con group (*p* = 0.0391), despite not being significant, and the NB group was lower than the PB group; there was no difference between the Con group and PB group (Figure 1C). The β-diversity analysis can reflect the coefficient of variation between samples, which can be visualized by principal coordinate analysis (PCoA). The sample points of the PB group were relatively concentrated and far away from the distribution of the remaining two groups (Figure 1D). These data demonstrated that intranasal transplantation of fecal microbiota led to disorganized nasal microbiota in mice. While PCoA separated PB from the controls, the diversity indices (Observed-otus/Chao1/Shannon) showed overlap, suggesting taxonomic shifts without overall diversity loss.

**Figure 2:**
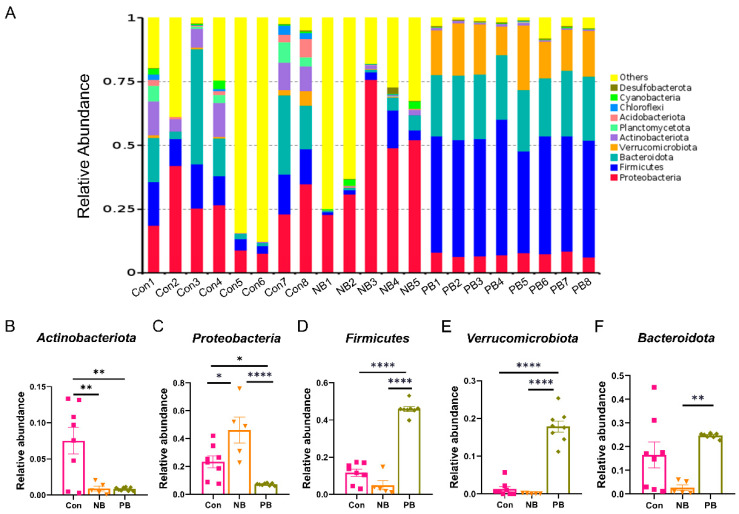
Differences in the abundance of nasal microbiota by groups at the phylum level. (**A**) Column cumulative plot of the relative abundance of species at the phylum level for each group of nasal microbiota. (**B**) Relative abundance of *Actinobacteriota*. (**C**) Relative abundance of *Proteobacteria*. (**D**) Relative abundance of *Firmicutes*. (**E**) Relative abundance of *Verrucomicrobiota.* (**F**) Relative abundance of *Bacteroidota*. Data are expressed as the mean ± SEM, and significance was tested using one-way ANOVA with Tukey’s post hoc test. * *p* < 0.05, ** *p* < 0.01 and **** *p* < 0.0001 (n = 5–8/group).

**Figure 3:**
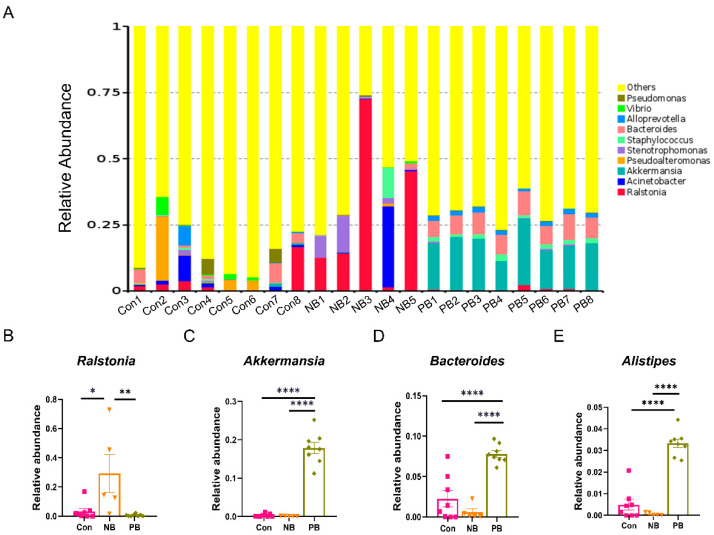
Differences in the abundance of nasal microbiota by groups at the genus level. (**A**) Column cumulative plot of the relative abundance of species at the genus level for each group of mice nasal microbiota. (**B**) Relative abundance of *Ralstonia*. (**C**) Relative abundance of *Akkermansia*. (**D**) Relative abundance of *Bacteroides.* (**E**) Relative abundance of *Alistipes*. Data are expressed as the mean ± SEM, and significance was tested using one-way ANOVA with Tukey’s post hoc test. * *p* < 0.05, ** *p* < 0.01 and **** *p* < 0.0001 (n = 5–8/group).

**Figure 4:**
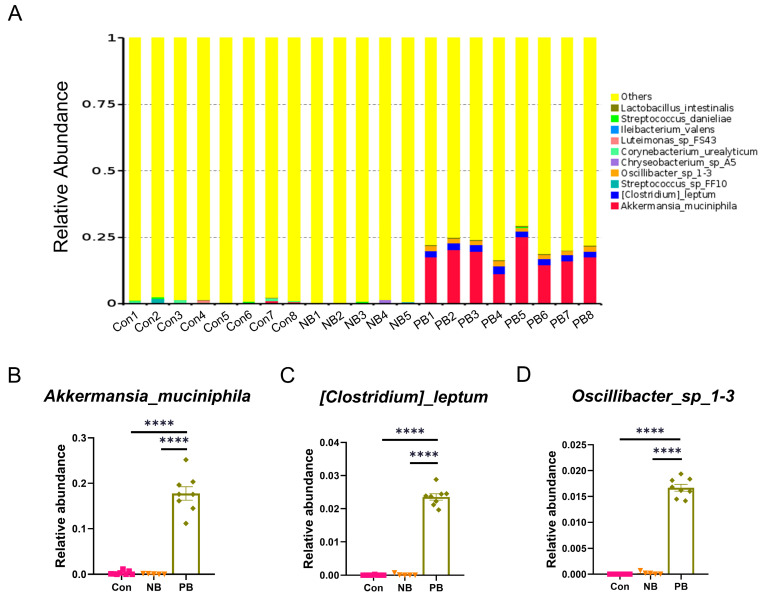
Differences in the abundance of nasal microbiota by groups at the species level. (**A**) Column cumulative plot of the relative abundance of species at the species level for each group of mice nasal microbiota. (**B**) Relative abundance of *Akkermansia_muciniphila*. (**C**) Relative abundance of *[Clostridium]_leptum*. (**D**) Relative abundance of *Oscillibacter*_sp_*1-3*. Data are expressed as the mean ± SEM, and significance was tested using one-way ANOVA with Tukey’s post hoc test. **** *p* < 0.0001. (n = 5–8/group).

We further analyzed the top 10 species in terms of abundance at the phylum, genus and species levels, respectively, and plotted them as a stacked bar chart of relative abundance of species (Figure 2, Figure 3 and Figure 4).

At the phylum level (Figure 2), the three groups showed quite significant differences in relative abundance (Figure 2A). The levels of *Actinobacteriota* were significantly lower in the PB and NB groups than those in the Con group (vs. NB *p* = 0.0063 and vs. PB *p* = 0.0020), and there was no difference between the PB and NB groups (Figure 2B). The levels of *Proteobacteria* were also significantly lower in the PB group than in the other two groups (vs. Con *p* = 0.0418 and vs. NB *p* < 0.0001) but showed a significant increase in the NB group compared to the others (*p* = 0.0114, Figure 2C). In contrast, the levels of *Firmicutes* and *Verrucomicrobiota* were significantly higher in the PB group than the other two groups (*p* < 0.0001); there was no difference between the Con and the PB groups (Figure 2D,E). For the levels of *Bacteroidota*, it was lower in the NB group, but the significant difference was only between the NB and the PB groups (*p* = 0.0024, Figure 2F).

At the genus level (Figure 3), significant alterations were also observed across the three groups (Figure 3A). The level of *Ralstonia* was significantly higher in the NB group than in the other groups (vs. Con *p* = 0.0128 and vs. PB *p* = 0.0062); there was no difference between the Con and the PB groups (Figure 3B). The levels of *Akkermansia*, *Bacteroidota* and *Alistipes* were significantly higher in the PB group than in the other two groups (*p* < 0.0001), while there was no difference between the other two groups (Figure 3C–E).

At the species level (Figure 4), the PB group showed great difference compared to the other two groups (Figure 4A). The levels of *Akkermansia_muciniphila*, *[Clostridium]_leptum* and *Oscillibacter*_sp_*1-3* were significantly higher in the PB group than in the others (*p* < 0.0001), and there was no difference between the Con and the NB groups (Figure 4B–D).

In summary, the intranasal transplantation of fecal microbiota altered the diversity and composition of the nasal microbiota in mice, and the PD-derived microbiota transplantation significantly altered the nasal bacterial composition at multiple levels.

### 3.2. Nasal Microbiota Dysbiosis-Induced Olfactory and Motor Dysfunction

**Figure 5:**
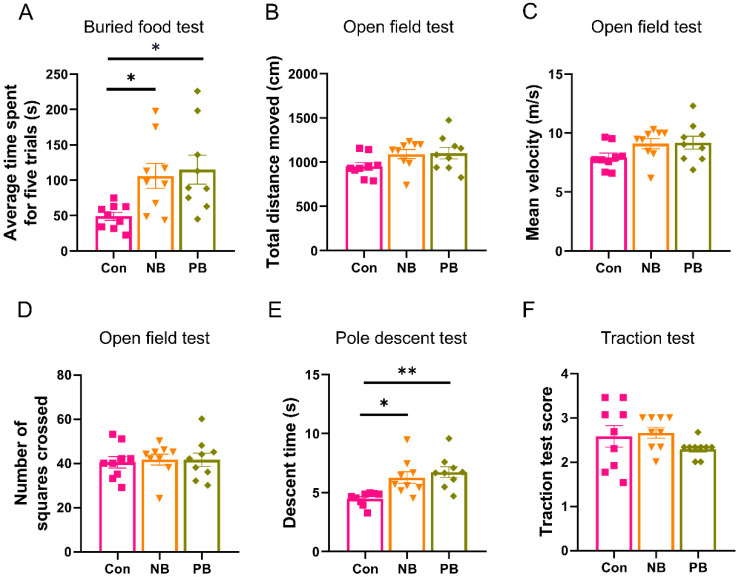
Nasal microbiota dysbiosis induced olfactory and motor dysfunction. (**A**) The time required for each group of mice in the buried food test, with the vertical coordinate indicating the average time of five experiments after removing the maximum and minimum values. (**B**–**D**) Open field test for each group of mice: (**B**) the total distance moved; (**C**) the mean velocity; (**D**) the number of squares crossed. (**E**) Pole descent test: The vertical coordinate is the average of the time taken by mice to climb down from the top of the rod in three experiments. (**F**) Traction test: The vertical coordinate is the mean of the traction test score in the three experiments. Data are expressed as the mean ± SEM, and significance was tested using one-way ANOVA with Tukey’s post hoc tests. * *p* < 0.05 and ** *p* < 0.01 (n = 9/group).

In order to test the effect of an intranasal microbiota transplant on the olfactory function of mice, we conducted the buried food experiment. The results showed that the average time required was higher in the nasal microbiota dysbiosis groups compared to the Con group (vs. NB *p* = 0.0464 and vs. PB *p* = 0.0195). There was no difference between the two nasal microbiota dysbiosis groups (Figure 5A). The above results suggested that nasal microbiota dysbiosis may disturb the olfactory function of mice.

In order to detect the effect of nasal microbiota dysbiosis on the motor function of mice, the open field test, pole descent test and traction test were used (Figure 5B–F). In the open field test, there was no difference at the total distance, the mean velocity and the number of squares crossed between these three groups (Figure 5B–D), which suggested that the present nasal microbiota dysbiosis did not affect the anxiety-like behavior. We also carried out the pole descent test and traction test, which are usually used for assessing coordination and neuromuscular functions in PD animal models [36]. In the pole descent test, nasal microbiota dysbiosis groups had a longer decent time compared to the Con group (vs. NB *p* = 0.0118 and vs. PB *p* = 0.0017), and there was no significant difference between the two nasal microbiota dysbiosis groups (Figure 5E). In the traction test, we did not find a significant difference between the three groups (Figure 5F). These results suggested that nasal microbiota dysbiosis also led to deficits in movements.

### 3.3. Neuronal Loss and Astrocyte Activation in the Olfactory-Related Brain Regions of the Mice with Nasal Microbiota Dysbiosis

To verify whether the nasal microbiota dysbiosis would cause the pathological changes in the brain, immunostaining and WB was done in the olfactory-related structures, including OB, PC and hippocampus.

**Figure 6:**
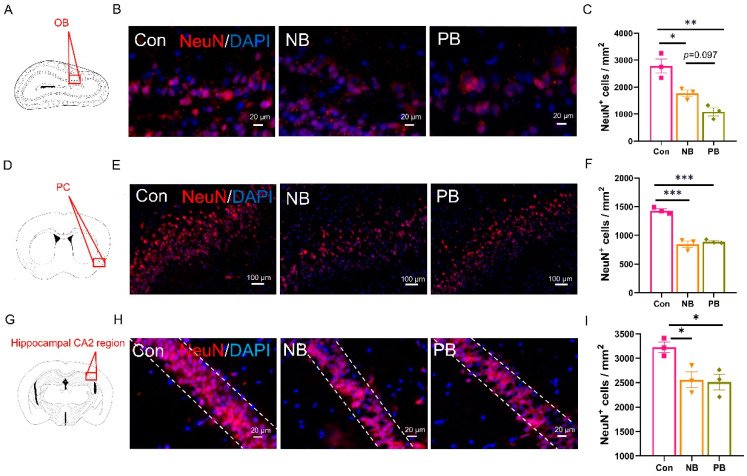
Neuronal loss in the olfactory-related brain regions in the nasal microbiota dysbiosis groups. (**A**) Schematic diagram of a coronal section of mouse OB, where the red box indicated the location in (**B**). (**B**) NeuN^+^ (red) IF results in the OB of each group; scale bar = 20 μm. (**C**) The number of NeuN^+^ cells in the OB of each group was shown as the cell density. (**D**) Schematic diagram of a mouse brain coronal section, where the red box indicated the location of PC in (**E**). (**E**) NeuN^+^ IF results in the PCs of each group; scale bar = 100 μm. (**F**) The number of NeuN^+^ cells in the PC of each group was shown as the cell density. (**G**) Schematic diagram of a mouse brain coronal section, where the red box indicated the location of the hippocampal CA2 region in (**H**). (**H**) NeuN^+^ IF results in the hippocampal CA2 region of each group; scale bar = 20 μm. (**I**) The number of NeuN^+^ cells in the hippocampal CA2 region of each group was shown as the cell density. Data are presented as the mean ± SEM, and significance was tested using one-way ANOVA with Tukey’s post hoc test. * *p* < 0.05, ** *p* < 0.01 and *** *p* < 0.001 (n = 3/group).

NeuN immunostaining was used to detect the neuronal loss (Figure 6). We found that, compared to the Con group, both nasal microbiota dysbiosis groups showed significant neuronal loss in OB (vs. NB *p* = 0.0207 and vs. PB *p* = 0.0017), PC (vs. NB *p* = 0.0001 and vs. PB *p* = 0.0002) and hippocampal CA2 region (vs. NB *p* = 0.0396 and vs. PB *p* = 0.0302, Figure 6A–I) but not in the hippocampal DG, CA1 and CA3 regions (Figure S1). There was no difference between the two nasal microbiota dysbiosis groups, except in the OB; the PB group showed a trend of more neuronal loss than NB group (*p* = 0.097, Figure 6C). These indicated that the nasal microbiota dysbiosis caused damage to neurons in the OB, PC and hippocampal CA2 region of mice, which might contribute to olfactory dysfunction in the NB and PB groups.

**Figure 7:**
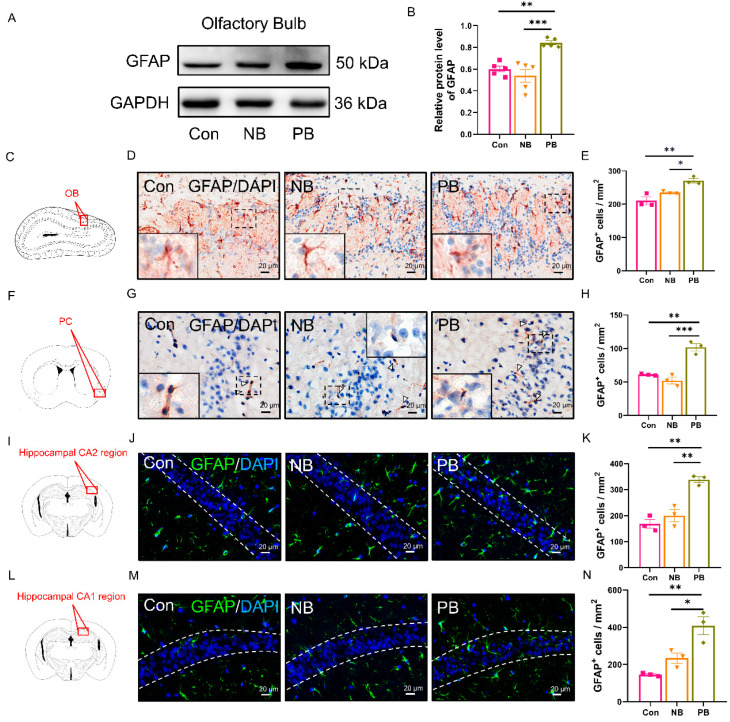
Detection of astrocytes activation in the olfactory-related brain regions of mice. (**A**) WB results of GFAP in the OB of each group. (**B**) Relative quantitative results of GFAP/GAPDH in the OB of each group (n = 5/group). (**C**) Schematic diagram of the mouse brain coronal section of OB, where the red box indicated the area in (**D**). (**D**) GFAP^+^ IHC results in the OB of each group. A magnified view of the area where the dashed box is located is shown in the lower left corner, which showed the morphology of an astrocyte. Scale bar = 20 μm. (**E**) The number of GFAP^+^ cells in the OB of each group was shown as the cell density. (**F**) Schematic diagram of a mouse brain coronal section, where the red box indicated the location of PC in (**G**). (**G**) GFAP^+^ IHC results in the PC of each group. Astrocytes have been marked out with white arrows. Upper right or lower left is a magnified view of the area where the corresponding dashed box is located, which shows the morphology of an astrocyte. Scale bar = 20 μm. (**H**) The number of GFAP^+^ cells in the PC of each group was shown as the cell density. (**I**) Schematic diagram of a mouse brain coronal section, where the red box indicated the location of the hippocampal CA2 region in (**J**). (**J**) GFAP^+^ IF results in the hippocampal CA2 region of each group. Scale bar = 20 μm. (**K**) The number of GFAP^+^ cells in the hippocampal CA2 region of each group was shown as the cell density. (**L**) Schematic diagram of a mouse brain coronal section, where the red box indicated the location of the hippocampal CA1 region in (**M**). (**M**) GFAP^+^ IF results in the hippocampal CA1 region of each group. Scale bar = 20 μm. (**N**) The number of GFAP^+^ cells in the hippocampal CA1 region of each group was shown as the cell density. Data are expressed as the mean ± SEM, and significance was tested using one-way ANOVA with Tukey’s post hoc test. * *p* < 0.05, ** *p* < 0.01 and *** *p* < 0.001 (n = 3/group).

The astrocyte activation was detected by the WB of GFAP or by the immunostaining of GFAP (the marker for the activation of astrocytes) and Iba-1 (the marker for the activation of microglia) (Figure 7). The results showed that, in all the other detected regions, the levels of GFAP were elevated only in the PB group compared to the other two groups, but there was no significant difference between the NB and Con groups (Figure 7A–N). In the hippocampus, the staining was still region-specific, which manifested as the increase in GFAP in the CA2 (vs. Con *p* = 0.0012 and vs. NB, *p* = 0.0036) and CA1 regions in the PB group (vs. Con *p* = 0.0027 and vs. NB *p* = 0.0195, Figure 7I–N), with no significant difference in the DG and CA3 regions between the three groups (Figure S2). We also examined the expression of Iba-1 in these brain regions, but no significant difference was detected among these experimental groups (Figure S3). We hypothesize that the present nasal microbiota dysbiosis were preferred to active astrocytes than microglia or astrocytes were first activated and then the microglia, since it was reported that microglia is activated following the activation of astrocytes [37,38].

Dopaminergic neuronal loss and astrocyte activation in the SN and striatum of the mice with nasal microbiota dysbiosis.

**Figure 8:**
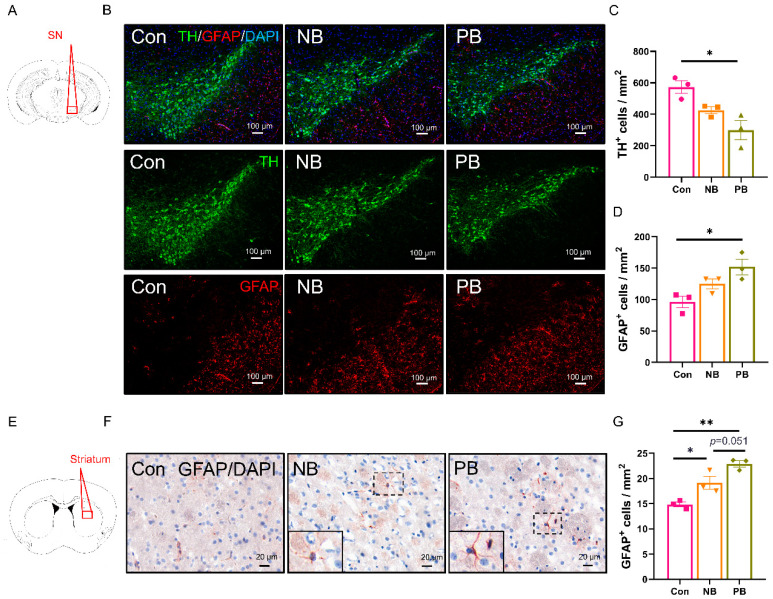
The detection of dopaminergic neuronal loss in the SN, and the astrocytes activation in the SN and the striatum. (**A**) Schematic diagram of a mouse brain coronal section, where the red box indicated the location of the SN in (**B**). (**B**) TH^+^ IF results in the SN of each group. Scale bar = 100 μm. (**C**) The number of TH^+^ cells in the SN of each group was shown as the cell density. (**D**) The number of GFAP^+^ cells in the SN of each group was shown as the cell density. (**E**) Schematic diagram of a mouse brain coronal section, where the red box indicated the location of the striatum in (**F**). (**F**) GFAP^+^ IHC results in the striatum of each group. The lower left corner is a magnified view of the area where the dashed box is located. Scale bar = 20 μm. (**G**) The number of GFAP^+^ cells in the striatum of each group was shown as the cell density. Data was presented as the mean ± SEM, and significance was tested using one-way ANOVA with Tukey’s post hoc test. * *p* < 0.05, ** *p* < 0.01 (n = 3/group).

Since PD specific microbiota intranasal transplantation induced behavioral disorder, PD-related brain regions of the SN and the striatum are adjacent to the olfactory-related brain regions such as PC and hippocampus anatomically, and we also examined the number of dopaminergic neurons and astrocytes in the SN or the striatum by immunostaining (Figure 8). In the SN, the decrease in TH^+^ cells (*p* = 0.0106) and the increase in GFAP^+^ cells in the SN were only observed in the PB group (*p* = 0.0181), and there was no significant difference between the Con group and the NB group (Figure 8A–D). In the striatum, compared to the Con group, both the nasal microbiota dysbiosis groups showed increased GFAP^+^ astrocytes (vs. NB *p* = 0.0294 and vs. PB *p* = 0.0015, Figure 8E–G). Despite GFAP^+^ astrocytes being more in the PB group than in the NB group, there was no significant difference between these two groups (*p* = 0.051, Figure 8G). The microglial activation in the SN and the striatum was detected by Iba-1 immunostaining, and there was no significant difference between these groups (Figure S4).

The above results indicated that nasal microbiota dysbiosis induced astrocyte activation and dopaminergic neuronal death in the SN or the striatum, which might be related to the motor dysfunction.

## 4. Discussion

Sequencing of 16S rRNA revealed that intranasal transplantation of fecal bacteria did change the composition of the original nasal microbiota of mice, and the PB group showed the greatest difference from the other two groups: (1) At the phylum level, there was a significant increase in the relative abundance of *Firmicutes* and *Verrucomicrobiota* and a significant decrease in the relative abundance of *Actinobacteriota* and *Proteobacteria* in the PB group. This was also observed in fecal samples from PD patients, as well as PD animal models [39,40,41,42,43]. In addition, *Verrucomicrobia* was reported to be associated with elevated plasma IFN-γ concentrations in PD patients [39]. (2) At the genus level, the relative abundance of *Akkermansia*, *Bacteroides* and *Alistipes* was significantly increased in the PB group. Highly enriched *Alistipes* has been shown to be positively correlated with the expression of TNF [44]; elevated relative abundance of *Bacteroides* was also correlated with the elevated plasma TNF-α and dyskinesia in PD patients [39]. For *Akkermansia*, it has been reported to increase in the fecal samples from PD patients and rotenone-induced PD animal models [39,40]. It has been shown to be significantly positively correlated with the level of LPS in the colon and negatively correlated with the number of TH^+^ cells in the SN [40]. (3) At the species level, the relative abundance of *Akkermansia_muciniphila*, *[Clostridium]_leptum* and *Oscillibacter_sp_1-3* was significantly increased in the PB group, where an increase in the amount of *Akkermansia_muciniphila* was also detected in fecal samples from PD animal models [42,45]. To date, *Akkermansia* is generally accepted as a beneficial bacterium [46]; however, it was also found to promote mucin degradation to exacerbate intestinal inflammation and permeability, leading to elevated endotoxemia and systemic inflammation. Notably, degradation of mucin by *Akkermansia_muciniphila* may also lead to a compensatory increase in mucin synthesis, which may, in turn, exert an anti-inflammatory effect in the host [45]. Thereby, the role of *Akkermansia_muciniphila* in PD needs further study. (4) Some putative proinflammatory bacteria such as *Ralstonia*, which abundance was elevated in PD patients’ colonic mucosa [47] but decreased in our intranasal transplanted animals, may be due to the altered environment from gut to nose. (5) On the whole, the nasal microbiota dysbiosis in our study mainly manifested as an increase in inflammatory bacteria, which might contribute to local or systemic immune response and neuronal death.

In our study, the two nasal dysbiosis groups showed olfactory and motor dysfunction, accompanied by neuronal loss and astrocyte activation, which indicated that nasal dysbiosis not only induced the pathological change and malfunction in the nose but also in the CNS. Notably, microbes from PD mice seem to have no different impact on olfactory or motor function compared to microbes from normal mice. One possible reason is that microbes derived from PD and normal mice may share certain microbial groups or metabolic characteristics. For example, animals received fecal bacteria from PD and normal mice showed similarity in an abundance of nasal microbiota in *Actinobacteriota* at the phylum level (Figure 2B). Another possibility is that the transplantation protocol details, including the dose of transplanted bacteria, the transplantation duration and the sampling time point, might also influence the outcomes. The protocol used in this study might not be sufficient to induce a significant behavioral difference between these two groups.

We found that the neuronal loss and astrocyte activation induced by nasal microbiota dysbiosis could ascend along the olfactory pathway and its adjacent structures, including the OB, PC, hippocampus, SN and striatum, so we speculated that astrocyte activation along the olfactory pathway might be the axis in the nose-to-brain crosstalk. Especially in the hippocampus, neuronal damage was only observed in the CA2 region, and astrocyte activation was only observed in the CA2 and CA1 regions in the PB group. As is well known, the hippocampus is associated with olfactory-related memory formation and contextualization, especially the hippocampal CA2 region, which has been reported to play a major role in regulating social recognition memory [48,49,50], which means nasal microbiota dysbiosis may cause the impairment of olfactory-related social recognition memory. In addition, interestingly, we also found a gradual attenuation of astrocyte activation and neuronal death from the OB, the PC and the hippocampus to the SN and the striatum (Supplemental Table S1), which is consistent with the clinical manifestations of olfactory disorders (related to the OB and the PC [51,52]) in the early stage [6] and cognitive disorders (related to the hippocampus [53,54]) and motor disorders (related to the SN and striatum [55,56]) in the late stage in PD patients [6].

As a pilot study, our findings provided evidence for the hypothesis of the microbiota–nose–brain axis, which also suggested that nasal microbiota should be an ideal target for the early diagnosis, for monitoring the disease progression and even for the therapy of PD or the other degenerative diseases. Especially, intranasal probiotics application holds promise as a novel strategy for the treatment or prevention of not only olfactory dysfunction but also degenerative disease. For example, *Lactobacillus casei* AMBR2 has been shown to downregulate the IL-1β, IL-8 and TNF-α levels in respiratory epithelial cells, alleviate inflammatory responses and inhibit the growth of nasal pathogenic bacteria [57]. Still, as a pilot investigation, the present study has its shortcomings and limitations. Larger-scale validation cohorts and expanded mechanistic studies are needed to establish causal relationships. In a future study, we believe that transplantation of nasal microbiota from PD animal models or patients is ideal. In addition, the trigeminal pathway has been suggested to be an important bridge in the nose–brain axis, so it should also be investigated. In addition, to minimize hormonal confounders, this study only focused on male mice, which is also a limitation. Since clinical evidence showed sexual dimorphism in PD onset and progression [58], further study on both sexes is needed.

## 5. Conclusions

In conclusion, our study found that the intranasal transplantation of pathogenic bacteria led to olfactory and motor dysfunction in mice, which might be due to the neuronal damage and astrocyte activation along or adjacent to the olfactory-related brain regions. Therefore, the microbiota–nose–brain axis should be seriously considered in any stage of PD or other degenerative diseases.

## Data Availability

The data that support the findings of this study are available from the corresponding author upon reasonable request. The data are not publicly available due to privacy.

## Supplementary Materials

The following supporting information can be downloaded at https://www.mdpi.com/article/10.3390/brainsci15050433/s1: Figure S1: NeuN staining in the hippocampal DG, CA3, CA1 sub-regions; Figure S2: GFAP staining in the hippocampal DG, CA3 sub-regions; Figure S3: Iba-1 staining in the olfactory related brain regions of mice; Figure S4: Iba-1 staining in the SN and the striatum of mice in each group; Table S1: Immunostaining results of astrocyte activation and neuronal loss in olfactory- and PD-related brain regions.
