# Supplementary material for: Intranasal Transplantation of Microbiota Derived from Parkinson’s Disease Mice Induced Astrocyte Activation and Neurodegenerative Pathology from Nose to Brain
Source: Brain Sci. 2025 Apr 23;15(5):433. doi: 10.3390/brainsci15050433 (PMC12109703; doi:10.3390/brainsci15050433)
Supplement: Supplementary file 1 [file brainsci-15-00433-s001.zip › brainsci-3574752-supplementary.pdf]

Supplementary Information

Supplementary Figures

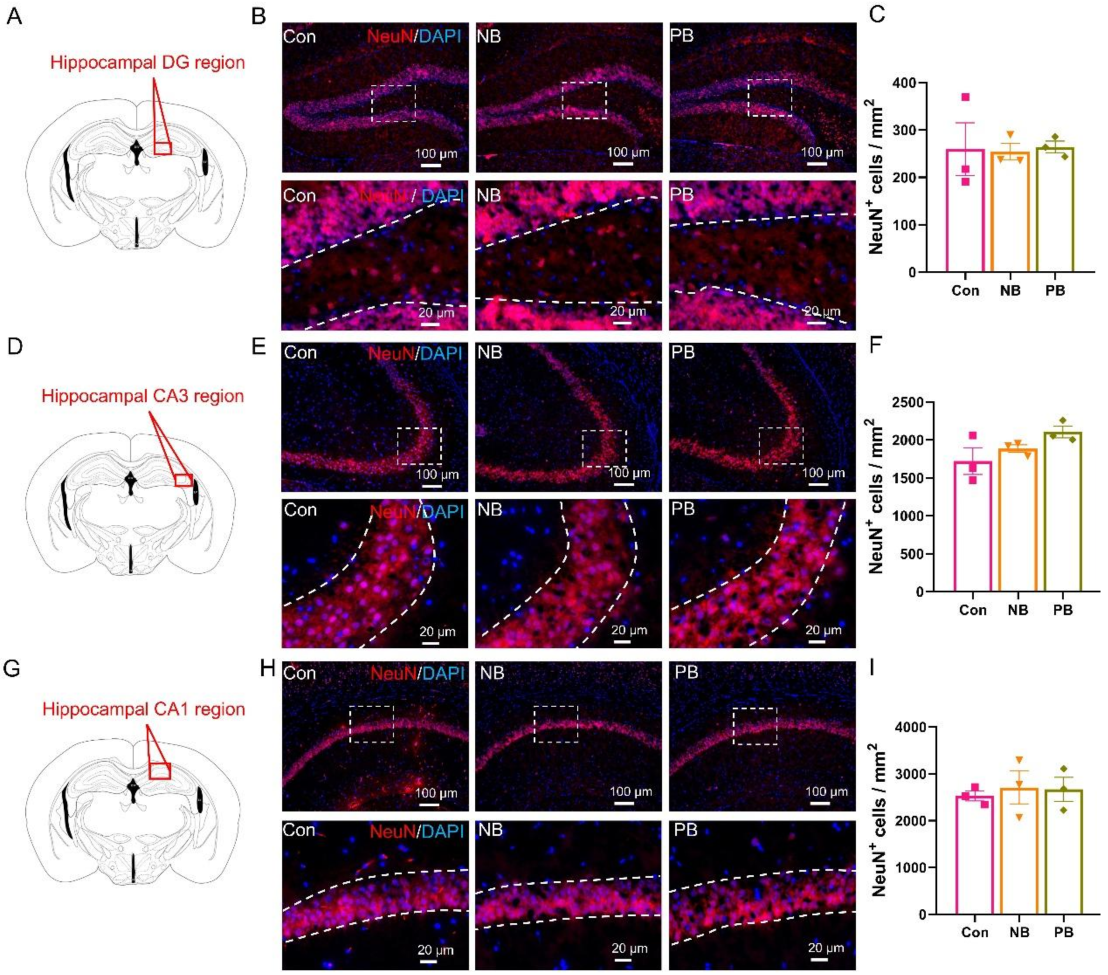

Figure S1. NeuN staining in the hippocampal DG, CA3, CA1 sub-regions. **A** Schematic diagram of mouse brain coronal section, where the red box indicated the location of hippocampal DG region in Fig. B. **B** NeuN$^+$ IF results in the hippocampal DG region of each group, scale bar = 100 μm. The lower figure is a magnified view of the dashed box area in the above figure, the dashed area indicated the location of hippocampal DG region, scale bar = 20 μm. **C** The number of NeuN$^+$ cells in the hippocampal DG region of each group was shown as cell density. **D** Schematic diagram of mouse brain coronal sections, where the red box indicated the location of hippocampal CA3 region in the Fig. E. **E** NeuN$^+$ IF results in the hippocampal CA3 region of each group, scale bar = 100 μm. The lower figure is a magnified view of the dashed box area in the above figure, the dashed area indicated the location of hippocampal CA3 region, scale bar = 20 μm. **F** The number of NeuN$^+$ cells in the hippocampal CA3 region of each group was shown as cell density. **G** Schematic diagram of mouse brain coronal sections, where the red box indicated the location of hippocampal CA1 region in the Fig. H. **H** NeuN$^+$ IF results in the hippocampal CA1 region of each group, scale bar = 100 μm. The lower figure is a magnified view of the dashed box area in the above figure, the dashed area indicated the location of hippocampal CA1 region, scale bar = 20 μm. **I** The number of NeuN$^+$ cells in the hippocampal CA1 region of each group was shown as cell density. Data are expressed as mean ± SEM, and significance was tested using one-way ANOVA with Tukey's post hoc test. (n = 3/group).

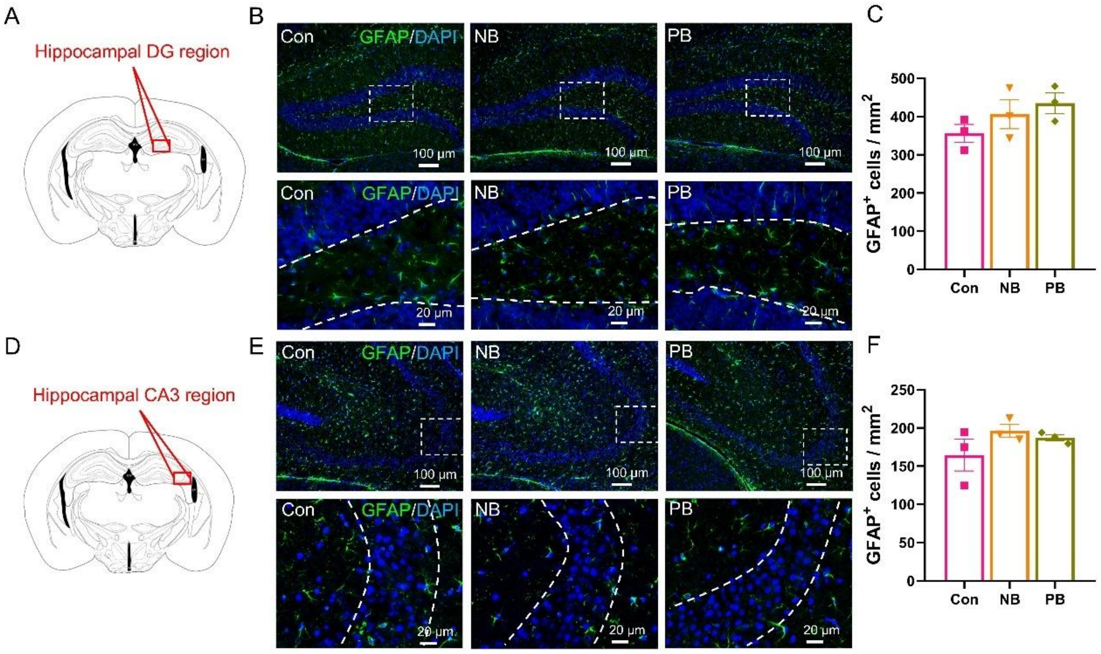

Figure S2. GFAP staining in the hippocampal DG, CA3 sub-regions. **A** Schematic diagram of coronal section of mouse brain, where the red box indicated the location of hippocampal DG region in Fig. B. **B** GFAP+ IF results in the hippocampal DG region of each group, scale bar = 100 μm. The lower figure is a magnified view of the dashed box area in the above figure, scale bar = 20 μm. **C** The number of GFAP+ cells in the hippocampal DG region of each group was shown as cell density. **D** Schematic diagram of mouse brain coronal sections, where the red box indicated the location of hippocampal CA3 region in the Fig. E. **E** GFAP+ IF results in the hippocampal CA3 region of each group, scale bar = 100 μm. The lower figure is a magnified view of the dashed box area in the above figure, scale bar = 20 μm. **F** The number of GFAP+ cells in the hippocampal CA3 region of each group was shown as cell density. Data are expressed as mean ± SEM, and significance was tested using one-way ANOVA with Tukey's post hoc test. (n = 3/group).

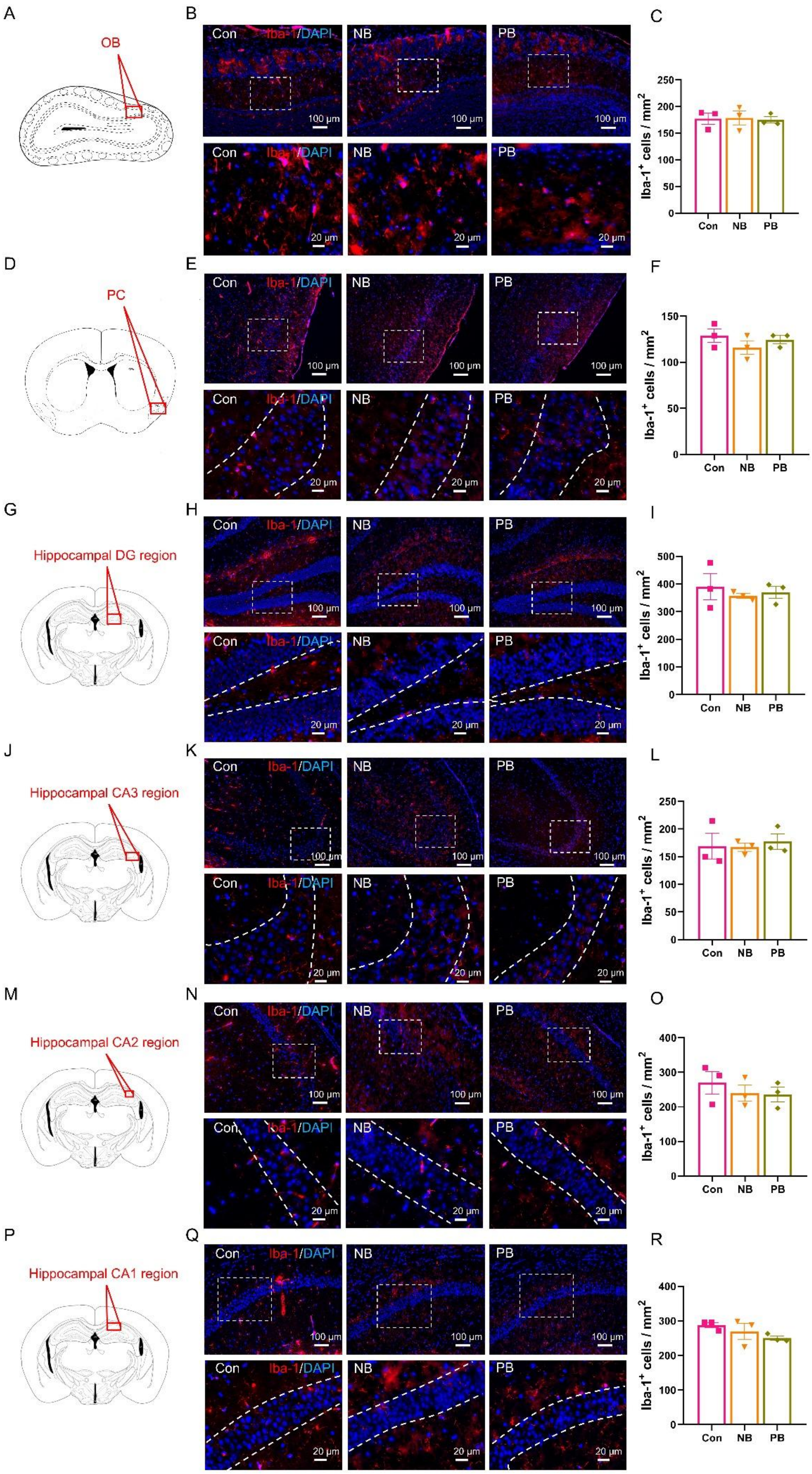

A
B
C
D
E
F
G
H
I
J
K
L
M
N
O
P
Q
R
OB
PC
Hippocampal DG region
Hippocampal CA3 region
Hippocampal CA2 region
Hippocampal CA1 region
Con
NB
PB
Iba-1/DAPI
100 μm
20 μm
Iba-1+ cells / mm2

Figure S3. Iba-1 staining in the olfactory related brain regions of mice. **A** Schematic diagram of coronal section of mouse brain, where the red box indicated the location of OB in Fig. B. **B** Iba-1^{+} IF results in the OB of each group, scale bar = 100 μm. The lower figure is a magnified view of the dashed box area in the above figure, scale bar = 20 μm. **C** The number of Iba-1^{+} cells in the OB of each group was shown as cell density. **D** Schematic diagram of coronal section of mouse brain, where the red box indicated the location of the PC in the E diagram. **E** Iba-1^{+} IF results in the PC of each group, scale bar = 100 μm. The lower figure is a magnified view of the dashed box area in the above figure, the dashed area indicated the location of PC, scale bar = 20 μm. **F** The number of Iba-1^{+} cells in the PC of each group was shown as cell density. **G** Schematic diagram of coronal section of mouse brain, where the red box indicated the location of the hippocampal DG region in the H diagram. **H** Iba-1^{+} IF results in the hippocampal DG region of each group, scale bar = 100 μm. The lower figure is a magnified view of the dashed box area in the above figure, Scale bar = 20 μm. **I** The number of Iba-1^{+} cells in the hippocampal DG region of each group was shown as cell density. **J** Schematic diagram of coronal section of mouse brain, where the red box indicated the location of the hippocampal CA3 region in the K diagram. **K** Iba-1^{+} IF results in the hippocampal CA3 region of each group, scale bar = 100 μm. The lower figure is a magnified view of the dashed box area in the above figure, Scale bar = 20 μm.**L** The number of Iba-1^{+} cells in the hippocampal CA3 region of each group was shown as cell density. **M** Schematic diagram of coronal section of mouse brain, where the red box indicated the location of the hippocampal CA2 region in the N diagram. **N** Iba-1^{+} IF results in the hippocampal CA2 region of each group, scale bar = 100 μm. The lower figure is a magnified view of the dashed box area in the above figure, Scale bar = 20 μm. **O** The number of Iba-1^{+} cells in the hippocampal CA2 region of each group was shown as cell density. **P** Schematic diagram of coronal section of mouse brain, where the red box indicated the location of the hippocampal CA1 region in the Q diagram. **Q** Iba-1^{+} IF results in the hippocampal CA1 region of each group, scale bar = 100 μm. The lower figure is a magnified view of the dashed box area in the above figure, Scale bar = 20 μm. **R** The number of Iba-1^{+} cells in the hippocampal CA1 region of each group was shown as cell density. Data are expressed as mean ± SEM, and significance was tested using one-way ANOVA with Tukey's post hoc test. (n = 3/group).

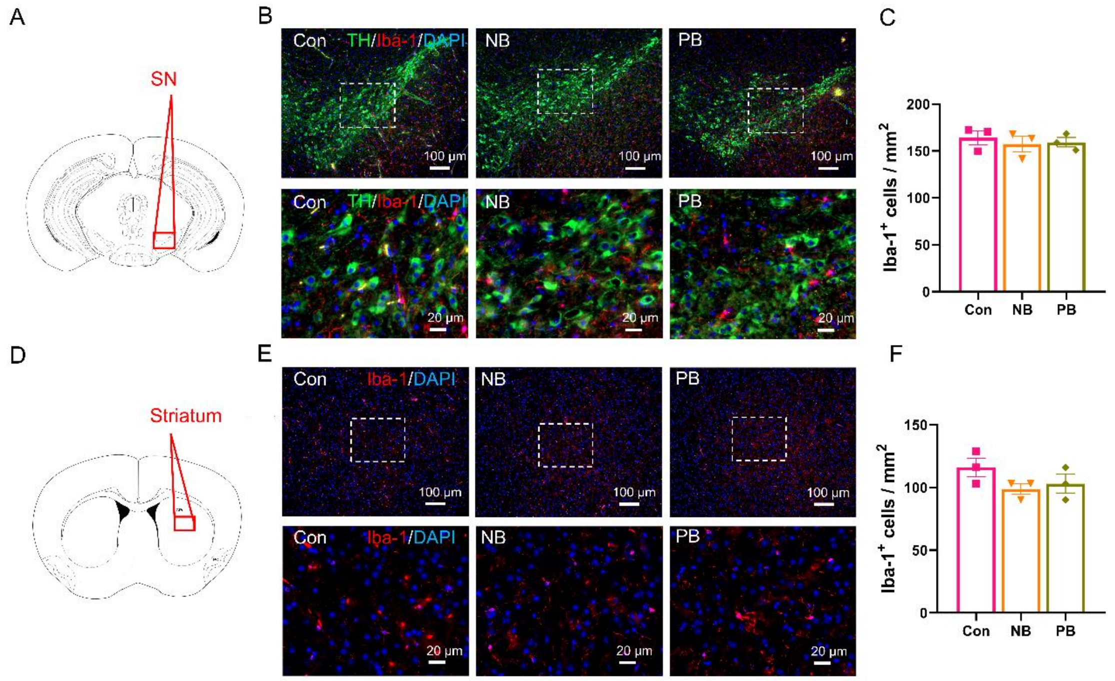

Figure S4. Iba-1 staining in the SN and the striatum of mice in each group. **A** Schematic diagram of coronal section of mouse brain, where the red box indicated the location of SN in Fig. B. **B** Iba-1^{+} IF results in the SN of each group, scale bar = 100 μm. The lower figure is a magnified view of the dashed box area in the above figure, scale bar = 20 μm. **C** The number of Iba-1^{+} cells in the SN of each group was shown as cell density. **D** Schematic diagram of coronal section of mouse brain, where the red box indicated the location of striatum in Fig. E. **E** Iba-

1+ IF results in the striatum of each group, scale bar = 100 μm. The lower figure is a magnified view of the dashed box area in the above figure, scale bar = 20 μm. **F** The number of Iba-1+ cells in the striatum of each group was shown as cell density. Data are expressed as mean ± SEM, and significance was tested using one-way ANOVA with Tukey's post hoc test. (n = 3/group).

Supplemental Table S1

Immunostaining results of astrocyte activation and neuronal loss in olfactory- and PD-related brain regions

	PB vs. Con		NB vs. Con		PB vs. NB	
	Astrocyte activation	Neuronal loss	Astrocyte activation	Neuronal loss	Astrocyte activation	Neuronal loss
OB	**	**	ns	*	*	ns
PC	**	***	ns	***	***	ns
Hippocampal CA2	**	*	ns	*	**	ns
Hippocampal CA1	**	ns	ns	ns	*	ns
Striatum	**	-	*	-	ns	-
SN	*	*	ns	ns	ns	ns
Hippocampal DG	ns	ns	ns	ns	ns	ns
Hippocampal CA3	ns	ns	ns	ns	ns	ns

Note. OB=Olfactory bulb, PC= Pyriform cortex, SN= Substantia nigra. *$P < 0.05$, **$P < 0.01$, ***$P < 0.001$, ns= non-significant, - = not detected.
